# Endothelium-Dependent Relaxation and Angiotensin II Sensitivity in Experimental Preeclampsia

**DOI:** 10.1371/journal.pone.0079884

**Published:** 2013-11-06

**Authors:** Anne Marijn van der Graaf, Marjon J. Wiegman, Torsten Plösch, Gerda G. Zeeman, Azuwerus van Buiten, Robert H. Henning, Hendrik Buikema, Marijke M. Faas

**Affiliations:** 1 Department of Obstetrics and Gynecology, University of Groningen, University Medical Centre Groningen, Groningen, The Netherlands; 2 Center for Liver, Digestive and Metabolic Diseases, Laboratory of Pediatrics, University of Groningen, University Medical Centre Groningen, Groningen, The Netherlands; 3 Department of Clinical Pharmacology, University of Groningen, University Medical Centre Groningen, Groningen, The Netherlands; 4 Division of Medical Biology, Department of Pathology and Medical Biology, University of Groningen, University Medical Centre Groningen, Groningen, The Netherlands; Otto-von-Guericke University Magdeburg, Germany

## Abstract

**Objective:**

We investigated endothelial dysfunction and the role of angiotensin (Ang)-II type I (AT1-R) and type II (AT2-R) receptor in the changes in the Ang-II sensitivity in experimental preeclampsia in the rat.

**Methods:**

Aortic rings were isolated from low dose lipopolysaccharide (LPS) infused pregnant rats (experimental preeclampsia; n=9), saline-infused pregnant rats (n=8), and saline (n=8) and LPS (n=8) infused non-pregnant rats. Endothelium-dependent acetylcholine--mediated relaxation was studied in phenylephrine-preconstricted aortic rings in the presence of vehicle, N^*G*^-nitro-L-arginine methyl ester and/or indomethacin. To evaluate the role for AT1-R and AT2-R in Ang-II sensitivity, full concentration response curves were obtained for Ang-II in the presence of losartan or PD123319. mRNA expression of the AT1-R and AT2-R, eNOS and iNOS, COX1 and COX2 in aorta were evaluated using real-time RT-PCR.

**Results:**

The role of vasodilator prostaglandins in the aorta was increased and the role of endothelium-derived hyperpolarizing factor and response of the AT1-R and AT2-R to Ang-II was decreased in pregnant saline infused rats as compared with non-pregnant rats. These changes were not observed during preeclampsia.

**Conclusion:**

Pregnancy induced adaptations in endothelial function, which were not observed in the rat model for preeclampsia. This role of lack of pregnancy induced endothelial adaptation in the pathophysiology of experimental preeclampsia needs further investigation.

## Introduction

Preeclampsia is a pregnancy specific syndrome, clinically characterized by the presence of hypertension, associated with proteinuria in the second half of pregnancy [1]. Preeclampsia complicates about 5% of pregnancies and is a leading cause of maternal and perinatal mortality [1]. The etiology of preeclampsia remains unknown, but appears to be related to the presence of the placenta [2]. In preeclamptic patients physiological remodelling of the uterine spiral arteries is diminished, resulting in decreased placental perfusion [3]. Several mechanisms have been implicated in the pathophysiology of preeclampsia, including activation of inflammatory cells [4], endothelial cell activation and vascular dysfunction [5], as well as changes in the renin-angiotensin-aldosterone system (RAAS) [6]. 

During normal pregnancy, vascular function changes dramatically; increased endothelium dependent vascular relaxation as well as increased flow mediated dilation can be observed [7]. Together this may result in a decrease in blood pressure (mainly in the second trimester) and a decrease in peripheral vascular resistance [8]. By production of vasoactive factors, endothelial cells are important mediators of vascular tone [9]. Changes in the production of these vasoactive factors may therefore account for the pregnancy-related changes in vascular relaxation. Indeed, the production of endothelial prostacyclin, nitric oxide (NO) as well as the unidentified endothelium-derived hyperpolarizing factor (EDHF) has been shown to be increased during pregnancy [10–13]. In contrast to normal pregnancy, vascular relaxation is reduced in preeclampsia [8]. The endothelial cell dysfunction in preeclampsia appears to be associated with an impaired regulation and secretion of vasodilating factors, such as NO, prostacyclin production or EDHF [12,14,15]. 

In addition, the RAAS may also be involved in the changes in vascular dysfunction in preeclampsia. While normal pregnancy is associated with a decreased sensitivity to the vasoconstrictor angiotensin II (Ang-II) [16], preeclampsia is associated with an increased response to Ang-II as compared to normal pregnancy [17]. During preeclampsia, the increased Ang-II sensitivity may even develop before the clinical manifestation of the disease [18,19]. Ang-II exerts its effects via two receptors. Binding of Ang-II to the Ang-II Type I receptor (AT1-R) causes contraction [17]. The other Ang-II receptor is the Type II receptor (AT2-R). The function of this receptor is less well understood. There is however, increasing evidence that the AT2-R may exert an inhibitory influence on AT1-R mediated stimulation [20]. It is largely unknown if and how these receptors are involved in the changes in Ang-II sensitivity during normal pregnancy and preeclampsia. However, it seems likely that the AT1-R is involved in the pathophysiology of preeclampsia, since in both rat and mice it has been demonstrated that treatment with AT1-R blockers inhibited the development of clinical signs in models of preeclampsia [21,22]. Unfortunately, treatment with AT1-R blockers is contraindicated during pregnancy [23].

In the present study, we evaluated endothelial function during pregnancy and experimental preeclampsia in the rat, by studying the role of the vasoactive factors in endothelial function as well as the role of the AT1-R and AT2-R in the Ang-II sensitivity. We used the well-established model for preeclampsia, i.e. the low-dose lipopolysaccharide (LPS) infused pregnant rat [24]. This model is characterized by hypertension and proteinuria and has been used as a model for preeclampsia for many years and was used in many studies [21,25–27], including a recent study by Wang et al.[28].

## Materials and Methods

### Animals

Experiments were conducted in accordance with the National Institutes of Health Guide for the Care and Use of Laboratory Animals and approved by the Committee for Animal Ethical Experiments of the University of Groningen (application number: DEC-5516A).

Female Wistar outbred rats (Harlan Inc, Horst, the Netherlands) were kept in a 12 hour light-dark cycle and constant room temperature, with food and water available ad lib in the home cages. Until selection for experiments vaginal smears were taken daily. Rats were rendered pregnant by housing them on pro-oestrus with fertile males for one night. Day 0 of pregnancy was documented by the presence of spermatozoa in the vaginal smear. In cyclic and pregnant rats, the latter ones on day 0 of pregnancy, a cannula was inserted into the right jugular vein under isoflurane/oxygen anesthesia according to standard methods [29]. The jugular vein cannula allows stress free infusion. On day 14 of pregnancy or 14 days after cannula placement, infusion of either saline or LPS took place. Day 14 of pregnancy was chosen since in the rat trophoblast invasion into the mesometrial triangle, i.e. the equivalent of the placental bed, and the spiral arteries starts around this day of pregnancy. 

The low-dose LPS treated pregnant rat is an established model of preeclampsia, characterized by hypertension, proteinuria, disseminated intravascular coagulation, generalized activation of the inflammatory response and endothelial cell activation [24,28,30]. LPS is infused at day 14 during 1h. The final concentration of LPS immediately after the infusion is found to be very low or even undetectable in some rats and from fifteen minutes onward undetectable in all rats (unpublished results). The development of the preeclamptic-like syndrome in this model is considered to result from a systemic inflammatory response induced by LPS [25,26,30]. In addition to studies focussing on the pathophysiology of preeclampsia, studies concerning therapeutic options for preeclampsia have also been performed in this model [27,28,31]. The rats were randomly divided into four groups as follows: non-pregnant (NP) saline infused (2 ml in 1 hour); pregnant (P) saline infused (2 ml in 1 hour); P LPS (E-Coli, 0.55: B5, Whittaker MA Bioproducts, Walkerville, Md.) infused (1μg/kg bw in 2 ml saline in 1 hour); NP LPS infused (1μg/kg bw in 2 ml saline in 1 hour). P-saline infused rats served as healthy pregnant controls whereas the LPS infused pregnant rats served as the preeclampsia group [24]. Six days after infusion (on day 20 of pregnancy), the animals were anesthetized with isoflurane/oxygen and decapitated. The aorta was isolated and placed in cold oxygenated Krebs solution. The number of pups was counted and their length as well as maternal weight measured.

### Drugs and chemicals

Krebs buffer (pH 7,4) was freshly made before the start of each experiment and contained in mmol/L: 120 NaCl, 5.9 potassium chloride (KCl), 25.2 NaHCO_3,_ 1.2 NaH_2_PO_4_, 10.4 glucose, 1.21 MgCl_2_·6H_2_O, and 2.52 CaCl_2_. All Krebs ingredients were purchased from E. Merck (Darmstadt, Germany). The stock solutions for phenylephrine (Sigma, St. Louis, MO, USA), acetylcholine (Sigma, St. Louis, MO, USA), Ang-II (Bachem AG, Bubendorf, Switzerland), PD-123319 (Park-Davis), Losartan (Merck Research laboratories, Rahway, USA), and N^*G*^-nitro-L-arginine methyl ester (L-NMMA; Calbiochem Brand of EMD Biosciences, Inc., La Jolla) were prepared in saline (0.9%NaCl in distilled water). Indomethacin (Sigma) was dissolved in NaHCO_3_.

### Aortic-ring contraction studies

The endothelium-dependent relaxation and sensitivity to Ang-II in aortic tissue was studied by standard isotonic contraction experiments with thoracic aorta rings of the rat as previously described [32,33]. Aortic rings (2mm) from the rats were kept in Krebs solution (at 37°C) and gassed with 95% CO_2_ and 5% O_2_. Prior to priming the aortic rings were equilibrated for 30 minutes and subsequently checked for viability by evoking a contraction with KCl (60mM) for 10 minutes. Excess aortic tissue was snap frozen and kept in -80°C.

### Endothelium-dependent relaxation

Eight aortic rings of each rat were used to study the endothelium-dependent relaxation. The rings were studied in duplo in the continuous presence of either vehicle, NO synthase inhibitor L-NMMA (10^-4^M), cyclooxygenase (COX) inhibitor indomethacin (10^-5^M) or with L-NMMA plus indomethacin, to study the resultant role of EDHF. After 20 minutes of pre-incubation, aortic rings were pre-contracted with 10^-6^M phenylephrine. Then, increasing concentrations of acetylcholine (10^-8^M - 10^-4^M) were added to the medium to investigate endothelium-dependent dilation after stabilization. Subsequently, the NO donor sodium nitroprusside (SNP; 10^-5^M) was added as a control for endothelium-independent relaxation. The mean acetylcholine -mediated relaxation of the two rings in each condition was calculated as a percentage of the phenylephrine mediated pre-contraction. 

### Response of the aortic rings to Ang-II

#### Functional response of the AT1-R to Ang-II

To determine the Ang-II induced contractile response via the AT1-R, two aortic rings of each rat were pre-incubated for 20 minutes with 10^-6^M PD-123319, an AT2-R antagonist [34] and the selective NO synthase inhibitor L-NMMA (10^-4^M) to prevent any confounding effects by the basal release of NO [35]. Both compounds were present during the entire experiment. Then, a cumulative Ang-II concentration-response curve (10^-10^M-10^-6^M) was obtained according to standard methods [36,37]. A subsequent amount of Ang-II was added after renewed stabilization. Following the response curve, a reference contraction response was evoked by stimulation with 10^-5^M phenylephrine and after stabilization of the phenylephrine response KCl (60mM) was added, to produce maximal contraction. The mean Ang-II-mediated contraction of the duplo rings in each condition was calculated as a percentage of the maximal KCl induced response. 

#### Functional response of the AT2-R to Ang-II

To determine the functional response of the AT2-R to Ang-II, two aortic rings of each rat were pre-incubated for 20 minutes with 10^-5^M losartan, an AT1-R antagonist [38]. This antagonist was present during the entire experiment. Following the pre-incubation period, the aortic rings were pre-contracted with 10^-6^M phenylephrine. After pre-contraction reached a stable contraction, cumulative Ang-II concentration-response curve (10^-10^M-10^-6^M) was obtained according to standard methods [36,37]. Thereafter, the NO donor SNP (10^-5^M) was added to the aortic rings, as a control for maximal endothelium-independent relaxation. For each rat, and for each dose of Ang-II, the mean Ang-II mediated vasodilatory response for the two rings was calculated as a percentage of the maximal pre-contraction with phenylephrine.

### Gene-expression analysis

Total aortic RNA was isolated with TriReagent (Sigma-Aldrich, St. Louis, MO) following the manufacturer’s instructions. Total RNA was quantified using a NanoDrop ND1000 spectrophotometer (NanoDrop Technologies Inc., Wilmington, DE). cDNA synthesis was performed as described before [39]. Real time quantitative PCR was performed using an Applied Biosystems 7900 FAST sequence detector (Foster City, California, USA) and Applied Biosystems reagents according to the manufacturer’s instructions. Expression levels were normalized to those of 18S ribosomal RNA which was analyzed in separate runs. Primers and probes for the AT1-R and AT2-R were obtained from Applied Biosystems (TaqMan Gene Expression Assays, AT1-R: Rn00578456_m1 and AT2-R: Rn00560677_m1). Primers and probes for iNOS, eNOS, COX1, COX2 and 18S ribosomal RNA were obtained from Invitrogen (Breda, Invitrogen). The sequences were (sense primer, antisense primer, and probe, respectively; all from 5’ to 3’): 18S (M11188), CGGCTACCACATCCAAGGA, CCAATTACAGGGCCTCGAAA, CGCGCAAATTACCCACTCCCGA. Cox1 (XM_579388.1), CCCAGAGTCATGAGTCGAAGG, AACAACAGGGATTGACTGGTGA, TTTCCCCTGCTGCTGCTCCTGC. Cox2 (NM_017232.2), TTGTTGAGTCATTCACCAGACAGAT, GCCTTTGCCACTGCTTGTACA, CCCCAGCAACCCGGCCAGC. Enos (NM_021838.2), AGGAAGTAGCCAATGCAGTGAA, AGCCATACAGGATAGTCGCCTT, CGCTTCGCCATCACCGTGCC. Inos (NM_012611), CTATCTCCATTCTACTACTACCAGATCGA, CCTGGGCCTCAGCTTCTCAT, CCCTGGAAGACCCACATCTGGCAG. 

The expression levels of AT1-R, AT2-R, iNOS, eNOS, COX1 and COX2 were normalized to those of 18S ribosomal RNA. 

### Statistics

Statistical analysis was performed using SPSS for Windows (Version 16.0), the EC_50_ and E_max_ were calculated using GraphPad Prism 5, on a standard computer. The independent sample T-test was used to analyze differences between the number and length of the rat pups. Two-way analysis of variance (Two-way ANOVA) was used to analyze differences in bodyweight of the rats. 

We show the dose response curves of all four groups after vehicle incubation. Whether there was a significant difference between the four groups of rats was tested using General Linear Model for repeated measures. To calculate whether NO, PG or EDHF played a significant role in the acetylcholine mediated relaxation the E_max_ of the different curves was analyzed and compared with the E_max_ of the vehicle curve (i.e. total relaxation) using Student *t*-test. To test differences in contribution of a certain factor between the four groups of rats, Two-way ANOVA was used. If the Two-way ANOVA detected significant differences, we tested whether P-saline infused rats differed from NP-saline infused rats and whether LPS infused rats differed from saline infused rats in both pregnant and non-pregnant groups, using independent student T-test with Bonferroni corrections. For the Ang-II response curves the E_max_ and EC_50_ were calculated to represent individual responses. Differences in the E_max_ and EC_50_ of the Ang-II response curves between the four groups were analyzed using Two-way ANOVA to detect an effect of pregnancy or LPS infusion. If the Two-way ANOVA detected significant differences, we tested whether P-saline infused rats differed from NP-saline infused rats and whether LPS infused rats differed from saline infused rats in both pregnant and non-pregnant groups, using independent student T-tests with Bonferroni corrections. To test whether correlations existed between Ang-II mediated relaxation through the AT2-R and the vasoactive role of NO, PG or EDHF in acetylcholine-mediated relaxation Pearson Correlation test were performed. PCR data are presented as relative gene expression to 18S ribosomalRNA and analyzed using Two-way ANOVA with log transformed data. We tested whether P-saline infused rats differed from NP-saline infused rats and whether LPS infused rats differed from saline infused rats in both pregnant and non-pregnant rats, using independent student T-test with Bonferroni corrections. In all cases, differences were considered significant if p≤0.05. Data are presented as mean ± SEM. 

## Results

The body weight was significantly increased in pregnant rats compared to non-pregnant rats (p<0.001). No significant effect of treatment (saline or LPS infusion) was found (p=0.093). The number of pups was not significantly different between the two pregnant groups (p=0.329). However, the length of the pups in the P-LPS infused rats was significantly lower compared to the P-saline rats (p=0.01). The number of resorptions did not differ between these groups, one animal of each group showed one resorption. Table 1 presents these rat characteristics.

**Table 1:** Rat characteristics.

**Group (n)**	**NP-saline (8)**	**P-saline (8)**	**P-LPS (9)**	**NP-LPS (8)**
Maternal weight (g)	242.12 (7.4)	325.50* (6,9)	344.22* (8.5)	249.75 (6.9)
Number of pups	-	12.0 (1.0)	13.44 (1.0)	-
Length pups (mm)	-	32.36^†^ (0.22)	31.57 (0.21)	-

P= pregnant; NP=non-pregnant; LPS= Lipopolysaccharide. Data are presented as mean ±SEM. n=number of rats in group. *: p<0.05 vs NP; ^†^ p<0.05 vs P-LPS.

### Endothelium-dependent relaxation in aortic rings

To study endothelial function in pregnancy and experimental preeclampsia in the rat, we used aortic rings, precontracted with phenylephrine and dilated with acetylcholine in the presence of vehicle, L-NMMA, to identify the role of NO, indomethacin, to identify the role of PG or L-NMMA and indomethacin, to identify the role of other factors then NO or PG, mainly EDHF. There was no effect of pregnancy or treatment (LPS or saline infusion), nor of the various inhibitors on the contraction with KCl prior to the start of the experiment (results not shown). Also, precontraction with phenylephrine following the incubation with the various inhibitors did not differ between the four groups, apart from the increased precontraction in P-saline rats vs the other three groups after indomethacin incubation (results not shown). Moreover, SNP evoked endothelial independent relaxation was decreased in P-saline rats vs NP-saline rats following incubation with all inhibitors (results not shown).

**Figure 1:**
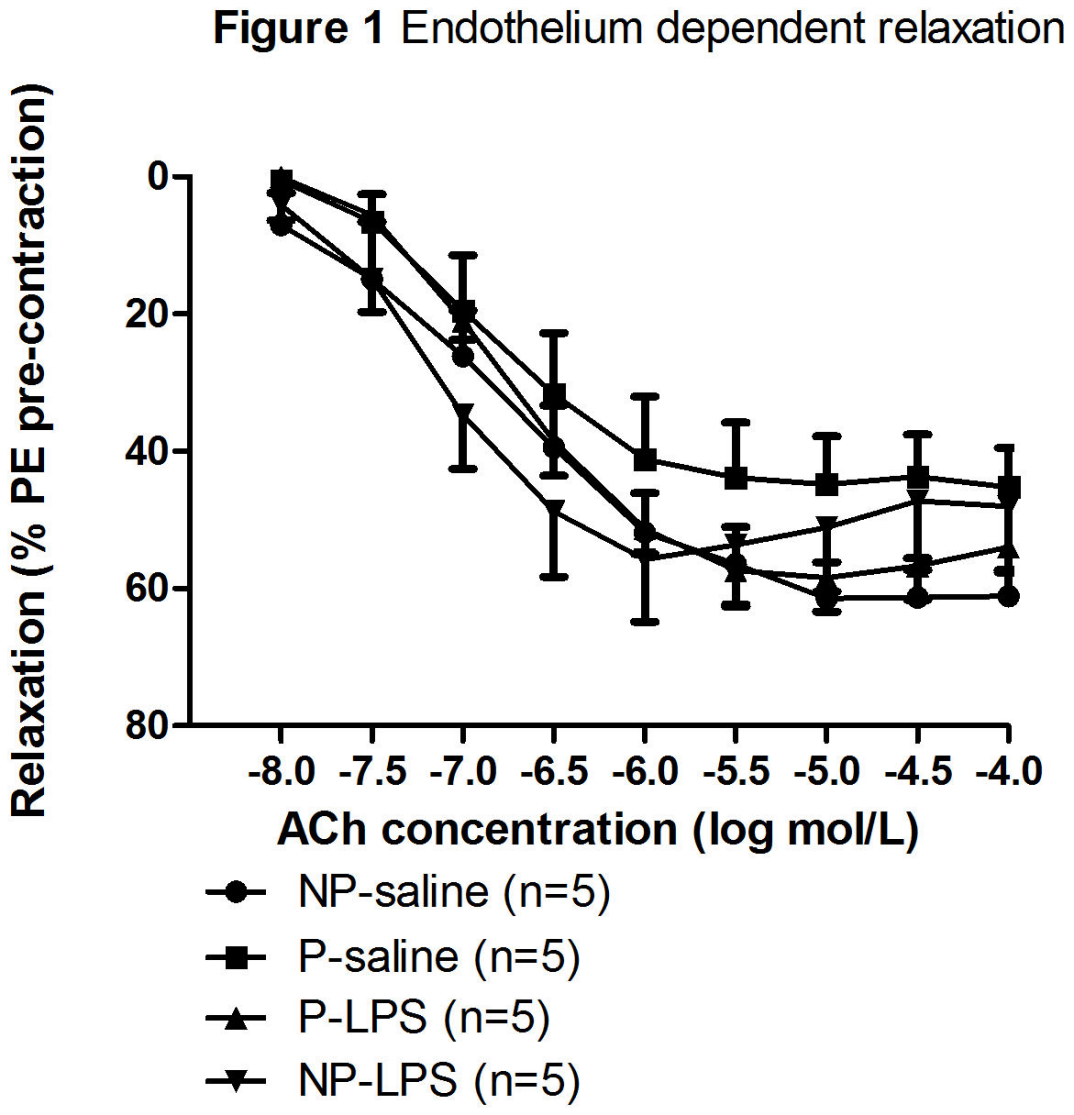
Endothelium dependent relaxation. The mean ± SEM acetylcholine-mediated endothelium dependent relaxation in the thoracic aorta of non-pregnant saline (NP-saline; circle), pregnant saline (P-saline; square), pregnant-LPS (P-LPS; triangle upward), and non-pregnant-LPS (NP-LPS; triangle downward) infused rats after incubation with vehicle. The percentage relaxation was calculated as percentage of the pre-contraction with phenylephrine (PE). Analyzing the data with General Linear Model of repeated measures showed no significant differences between the curves in the four groups.

**Figure 2:**
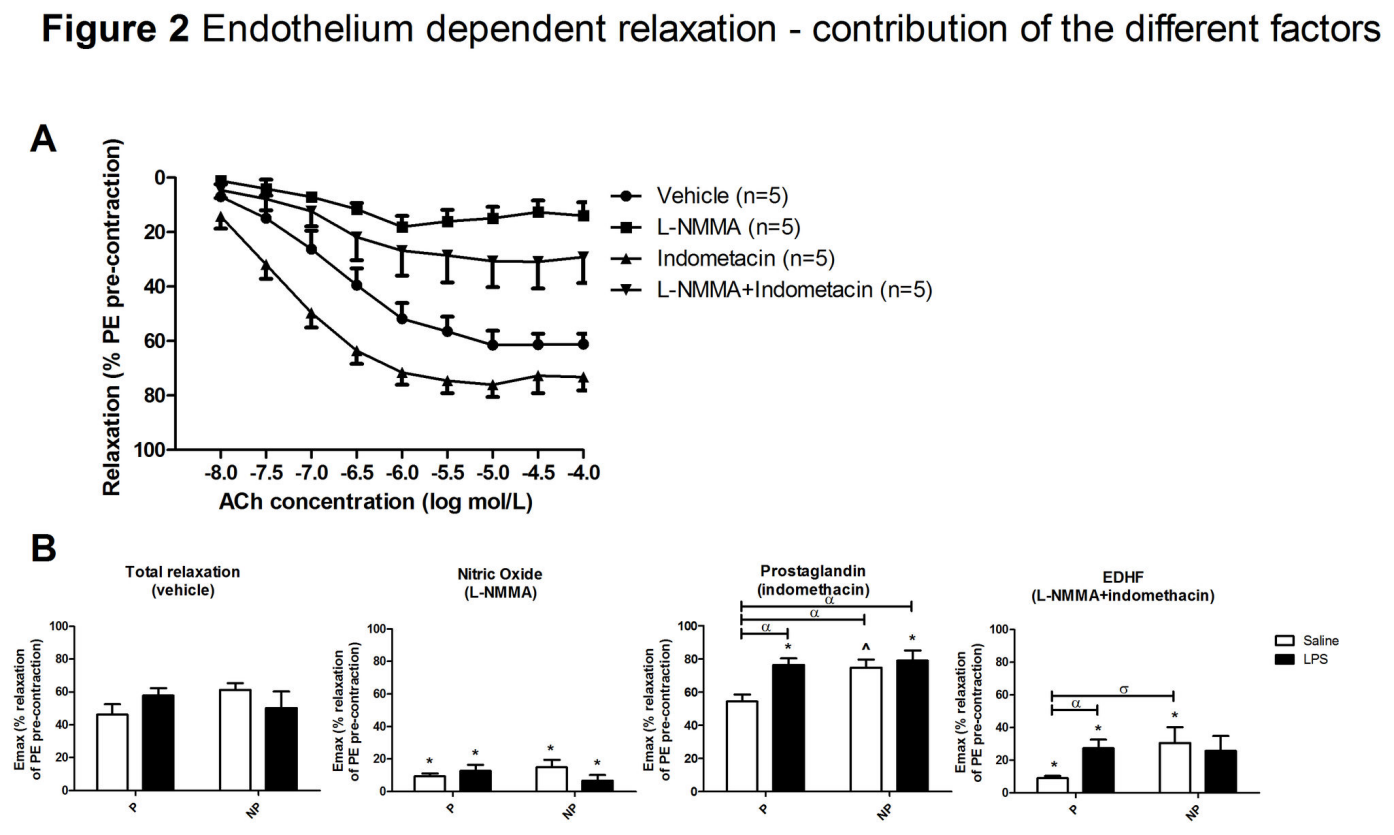
Endothelium dependent relaxation – contribution of the different factors. (**A**) The mean ± SEM acetylcholine-mediated endothelium dependent relaxation in the thoracic aorta of the non-pregnant saline infused rats after vehicle incubation (total relaxation; circle), after L-NMMA incubation (nitric oxide; square), after indomethacin incubation (prostaglandin; pyramid upward), and after L-NMMA and indomethacin incubation (EDHF; pyramid downward). The percentage relaxation was calculated as percentage of the pre-contraction with phenylephrine (PE). (**B**) The E_max_ of the endothelium dependent relaxation under the different conditions in the thoracic aorta from pregnant rats (P; left set of bars) and non-pregnant rats (NP; right set of bars) infused with saline (white bars) or lipopolysaccharide (LPS; black bars). Data are presented as mean ± SEM. *: p<0.05 vs E_max_ of the total relaxation within the same group of rats (Student *t*-test). α: p<0.05 vs P-saline; σ: p<0.1 vs P-saline (Two-way ANOVA). A trend towards a significant interaction between pregnancy and treatment (saline or LPS) was found for prostaglandin (p=0.09).

After vehicle incubation, all routes for relaxation are available and by adding acetylcholinein a cumulative fashion after precontraction with phenylephrine, relaxation appeared equal in all four groups (Figure 1). No significant difference in total acetylcholine mediated relaxation was seen between the four groups. Moreover, no significant differences in -logEC_50_ or E_max_ were found between the four groups (Table 2; acetylcholine relaxation and Figure 2B).

**Table 2:** logEC_50_ dose response curves.

**Group**	**NP-saline**	**P-saline**	**P-LPS**	**NP-LPS**
Acetylcholine relaxation (total relaxation)	6.82 ± 0.21	6.87 ±0.28	6.86 ± 0.06	6.63 ± 0.56
Acetylcholine relaxation in presence of L-NMMA	6.74 ± 0.29	6.58 ± 0.10	6.23 ± 0.52	6.79 ± 0.28
Acetylcholine relaxation in presence of Indomethacin	7.25 ± 0.13	6.98 ± 0.12	7.00 ± 0.16	7.24 ± 0.08
Acetylcholine relaxation in presence of L-NMMA + indomethacin	6.65 ± 0.14	6.69 ± 0.25	6.89 ± 0.17	6.45 ± 0.17
Ang-II contraction	7.92 ± 0.10*	7.56 ± 0.38	8.12 ± 0.11*	7.75± 0.10
Ang-II relaxation	8.33± 0.52	10.21 ± 3.74	9.45 ± 0.80	9.48 ± 0.40

P= pregnant; NP=non-pregnant; LPS= Lipopolysaccharide.

Two-way ANOVA showed no effect of pregnancy or treatment (saline or LPS infusion) for acetylcholine mediated relaxation, for L-NMMA, indomethacin and L-NMMA plus indomethacin, and for Ang-II induced relaxation. Two-way ANOVA did shown an interaction between pregnancy and treatment for Ang-II mediated contraction (p=0.0122). Therefore, Student T-tests were performed with Bonferroni corrections.

Data are presented as mean ± SEM. * p<0.001 vs P-saline.

The contribution of the different factors to the acetylcholine mediated relaxation is presented in Figure 2. Figure 2A shows the curves after vehicle, L-NMMA, indomethacin, and L-NMMA plus indomethacin incubation in NP-saline infused rats as an example. This figure shows that when aortic rings were relaxated in the presence of L-NMMA, which inhibits NO production, we observed a significantly decreased relaxation. This curve represents the resultant relaxation in the absence of NO. The curve after incubation with indomethacin, which inhibits the production of PG, represents the resultant relaxation in the absence of PG. In the presence of indomethacin, we observed a significant increase in relaxation as compared with the curve after vehicle incubation. This increase in relaxation following indomethacin indicates that the aorta produced mainly contractile PG (Figure 2A). When aortic rings were incubated with both L-NMMA and indomethacin, production of both NO and PG are inhibited, the curve therefore represents the resultant relaxation, which is due to other factors, i.e. EDHF. After incubation of aortic rings with L-NMMA and indomethacin, relaxation was significantly decreased as compared with the relaxation after vehicle incubation (Figure 2A). 

Figure 2B shows the E_max_ of the acetylcholine induced relaxation after incubation with vehicle, L-NMMA, indomethacin or L-NMMA and indomethacin of aortic rings of the four groups of rats. Since the E_max_ for NO and PG was calculated after incubation with their blockers (L-NMMA and indomethacin respectively), a higher E_max_ represents a lower NO or PG production. Since the curve after L-NMMA plus indomethacin incubation represents the relaxation due to EDHF, a higher E_max_ for this curve, represents a higher EDHF production. 

The total relaxation, i.e. relaxation after vehicle incubation, did not differ between the groups (Figure 2B, first graph). Also no difference in -logEC_50_ for total relaxation was observed between the groups (Table 2). After incubation of the aortic rings with L-NMMA, when NO production is inhibited, the E_max_ is very low and significantly decreased from the E_max_ after vehicle incubation in all groups, with no differences between the groups. This indicates that the resultant relaxation in the absence of NO is very small. NO thus play a large role in the relaxation of the aorta in all groups (Figure 2B, second graph). Also no difference in -logEC_50_ after L-NMMA incubation was observed between the groups (Table 2). After incubation with indomethacin, when PG production is inhibited, the E_max_ is significantly increased compared to the E_max_ of the vehicle curve, in NP-saline and LPS infused rats and in P-LPS infused rats (Figure 2B, third graph and Table 2). Thus in the absence of PG, the aorta’s showed an increased relaxation, indicating that in these aorta’s mainly contractile PG are produce. In P-saline infused rats, however, the E_max_ after indomethacin incubation was not different from the E_max_ following vehicle incubation, suggesting that PG are not produced by aorta of these pregnant rats. The E_max_ from P-saline infused rats is significantly lower than the E_max_ of the other three groups. However, no difference in -logEC_50_ after indomethacin incubation was observed between the groups (Table 2).

The last graph of Figure 2B shows the E_max_ after incubation with L-NMMA and indomethacin, i.e. when both NO and PG are inhibited. This E_max_ represents the relaxation due to other factors than NO and PG, i.e. EDHF. The resultant relaxation due to EDHF is low, indication a minor role for EDHF in the contraction of the aorta after acetylcholine and significantly decreased from the E_max_ after vehicle incubation in all groups except in the NP-LPS infused rats. However, the E_max_ after L-NMMA and indomethacin incubation was significantly increased in P-LPS infused rats as compared to the E_max_ of the P-saline infused rats, suggesting a more important role for EDHF in acetylcholine-induced aortic relaxation in P-LPS as compared to P-saline infused rats. However, no difference in -logEC_50_ after L-NMMA and indomethacin incubation was observed between the groups (Table 2).

These data thus show that pregnancy induced a shift in components inducing relaxation compared to NP rats, which is annihilated in preeclampsia.

#### Endothelium mRNA expression

**Figure 3:**
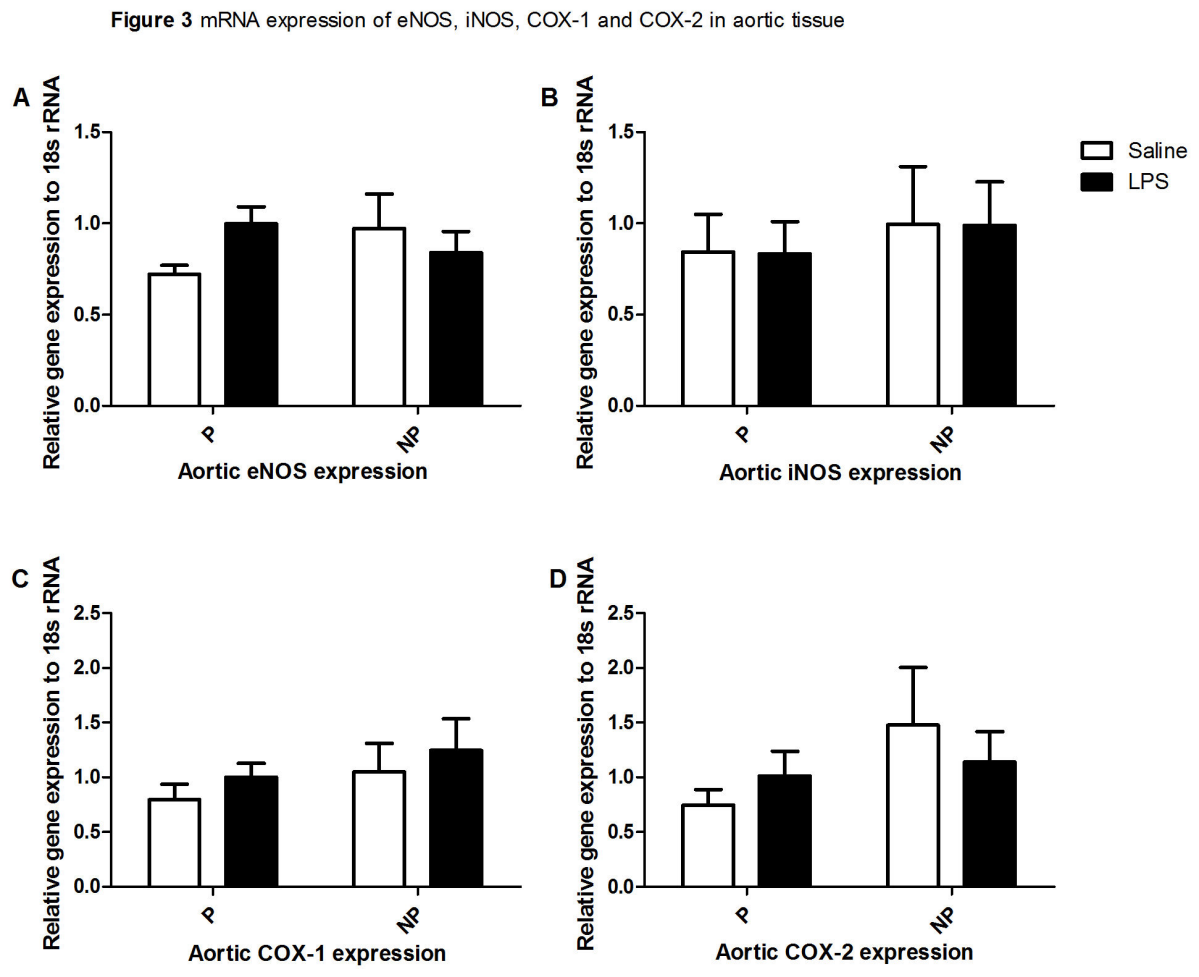
mRNA expression of eNOS, iNOS, COX-1 and COX-2 in aortic tissue. The mRNA expression of eNOS (**A**), iNOS (**B**), COX-1 (**C**) and COX-2 (**D**) in aortic tissue from pregnant-saline (left set of bars) and non-pregnant (right set of bars) infused with saline (open bars) or LPS (black bars). Two-way ANOVA showed no effect of pregnancy or treatment (saline or LPS infusion).

Four types of enzymes related to the production of NO and PG were analyzed for expression of their mRNA level in thoracic aortas. mRNA expression of endothelial and inducible nitric oxide synthase (eNOS and iNOS respectively) was not different between the four groups. Also, mRNA expression of COX-1 and COX-2 was not different between the four groups. Results are shown in Figure 3.

### Response of aortic rings to Ang-II

#### Ang-II mediated contraction

**Figure 4:**
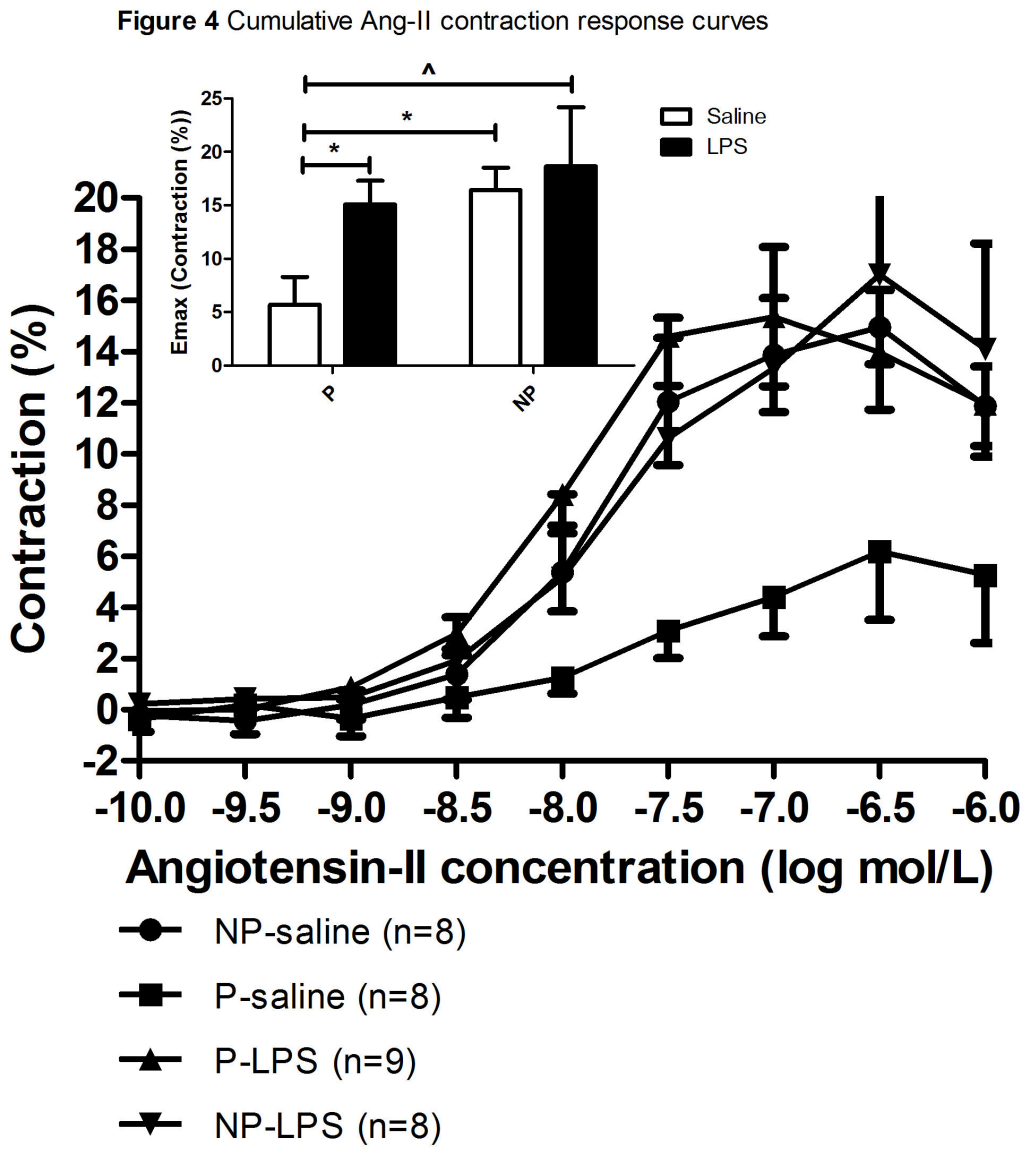
Cumulative Ang-II contraction response curves. The mean ± SEM cumulative Ang-II contraction curves in the thoracic aorta from the female rat in the non-pregnant-saline (NP-saline; circle; n=8), the pregnant-saline (P-saline; square; n=9), the pregnant-LPS (P-LPS; pyramid upward; n=9), and the non-pregnant-LPS (NP-LPS; pyramid downward; n=8) group. Percentages are calculated as percentage of the maximum contraction reached after adding 10^-5^M phenylephrine and 60mM KCl, at the end of the concentration response curves. Inset: Mean ± SEM E_max_ of the cumulative contraction curves. Two-way ANOVA showed a significant effect of pregnancy(p=0.05) and a trend for treatment (p=0.1), with no interaction effect between pregnancy and treatment (p=0.315). The effect of pregnancy and treatment was further analyzed with Student T-test using Bonferroni corrections. *: p<0.05; ^: p<0.1.

Figure 4 represents the cumulative Ang-II contraction curves (E_max_ shown in inset). In the presence of the AT2-R blocker PD-123319, when Ang-II can only bind to the AT1-R, contraction was observed in all groups. However, Ang-II mediated contraction was significantly blunted in P-saline infused rats (significantly decreased E_max_) compared to NP-saline infused rats (p=0.007). Moreover, after LPS infusion in P-rats a significant increase in Ang-II mediated contraction was seen as compared to the P-saline infused rats (p=0.017). There was, however, no effect of LPS treatment compared to saline infusion pertaining to the response of the AT1-R to Ang-II in NP-rats (p=0.713). Table 2 shows the -logEC_50_ of the Ang-II mediated contraction dose response curves. The -logEC_50_ was significantly increased in P-LPS infused rats as compared P-saline infused rats (p<0.001). Moreover, the -logEC_50_ was significantly increased in NP-saline infused rats as compared to P-saline infused rats (p<0.01). 

Thus, the pregnancy related decrease in Ang-II mediated contraction is absent in preeclampsia.

#### Ang-II mediated relaxation through the AT2-R

**Figure 5:**
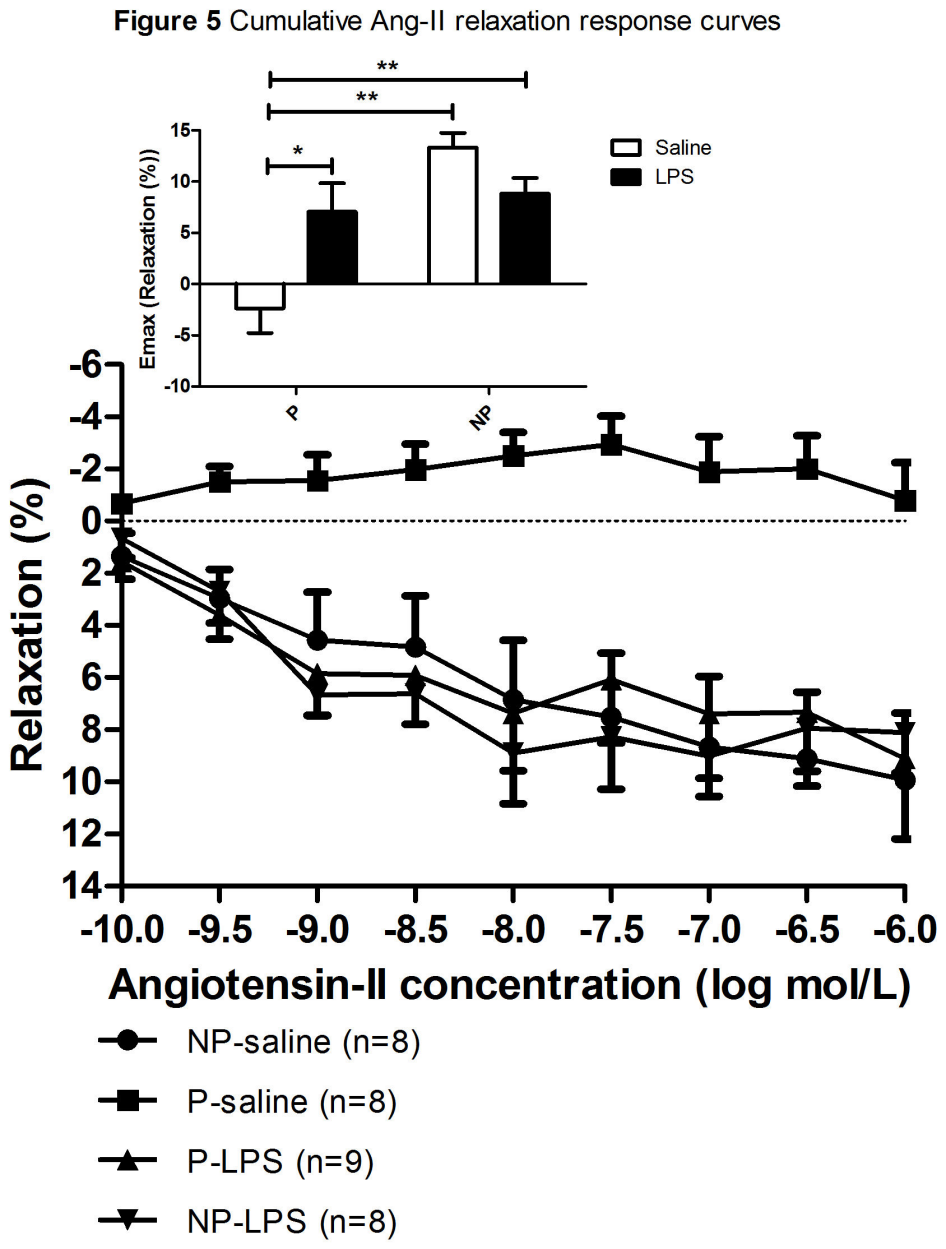
Cumulative Ang-II dilation response curves. The mean ± SEM cumulative Ang-II dilation curves of the in the thoracic aorta of non-pregnant saline (NP-saline; circle), pregnant saline (P-saline; square), pregnant-LPS (P-LPS; triangle upward), and non-pregnant-LPS (NP-LPS; triangle downward) infused rats. Percentages are calculated as percentage contraction upon Ang-II of the maximum contraction reached after adding 10^-6^M phenylephrine (PE). Inset: Mean ± SEM E_max_ of the cumulative Ang-II dilation curves. Two-way ANOVA showed a significant effect of pregnancy (p=0.001), with an interaction effect between pregnancy and treatment (p=0.006). The effect of pregnancy and treatment was further analyzed with Student T-test using Bonferroni corrections. *: p<0.05; **: p<0.01.

Figure 5 represents the cumulative Ang-II dilation response curves (E_max_ shown in inset). In the presence of the AT1-R blocker losartan, when Ang-II can only bind to the AT2-R, relaxation was observed in all but the P-saline infused rats. The response upon Ang-II (i.e. the E_max_) was significantly blunted in P-saline compared to NP-saline infused rats (p<0.001). Infusion of LPS in pregnant rats increased the Ang-II mediated dilation compared to P-saline infused rats (p=0.05). There was no effect of LPS on the Ang-II sensitivity in non-pregnant rats (p=0.06). No significant differences were found between the four groups in the -logEC_50_ of the Ang-II mediated relaxation through the AT2-R dose response curves (Table 2).

Thus, preeclampsia seems to reverse the decrease in Ang-II mediated relaxation through the AT2-R seen in healthy pregnancy compared to the non-pregnant situation.

#### Ang-II receptor expression

**Figure 6:**
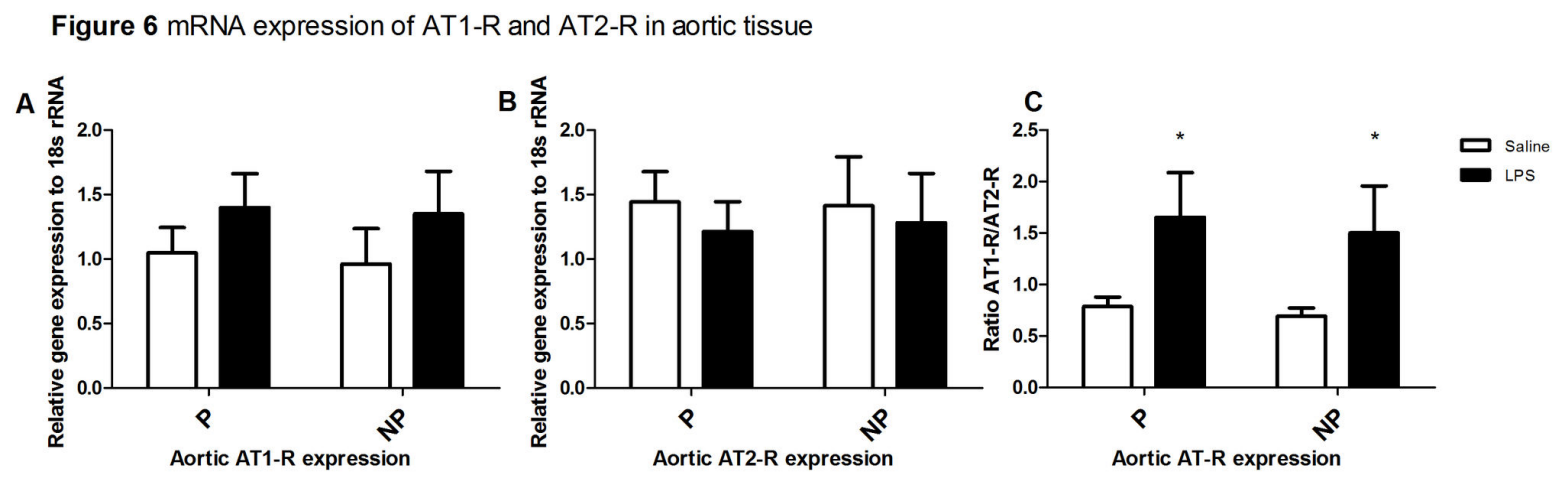
mRNA expression of AT1-R and AT2-R in aortic tissue. The mRNA expression of the AT1-R (**A**) and the AT2-R (**B**) in aortic tissue from pregnant-saline (left set of bars) and non-pregnant (right set of bars) infused with saline (open bars) or LPS (black bars). Values for AT1-R and AT2-R mRNA were normalized to those of 18S ribosomal RNA. Two-way ANOVA was used to analyze the data. No significant differences were found between either LP and NP, saline infusion and LPS infusion or the interaction effect between pregnancy and treatment, in aortic tissue. **C.**) The ratio of the AT1-R and AT2-R mRNA expression in aortic tissue from pregnant-saline (left set of bars) and non-pregnant (right set of bars) infused with saline (open bars) or LPS (black bars). Values for AT1-R and AT2-R mRNA were normalized to those of 18S ribosomal RNA. The ratio was calculated by dividing AT2-R to AT1-R. Using two-way ANOVA a significant effect of treatment (p=0.03) was found in aortic tissue, independent of pregnancy. *:p<0.05, LPS versus saline.

There was no effect of pregnancy or treatment (LPS or saline infusion) on AT1-R or AT2-R mRNA expression in aortic tissue. However, the ratio between the AT1-R and the AT2-R mRNA in aortic tissue showed a significant effect of treatment. The LPS-infused rats showed a significant higher ratio (Figure 6) compared to the saline-infused rats, regardless of pregnancy (p=0.03).

### Correlation endothelium-dependent relaxation and response to Ang-II

**Figure 7:**
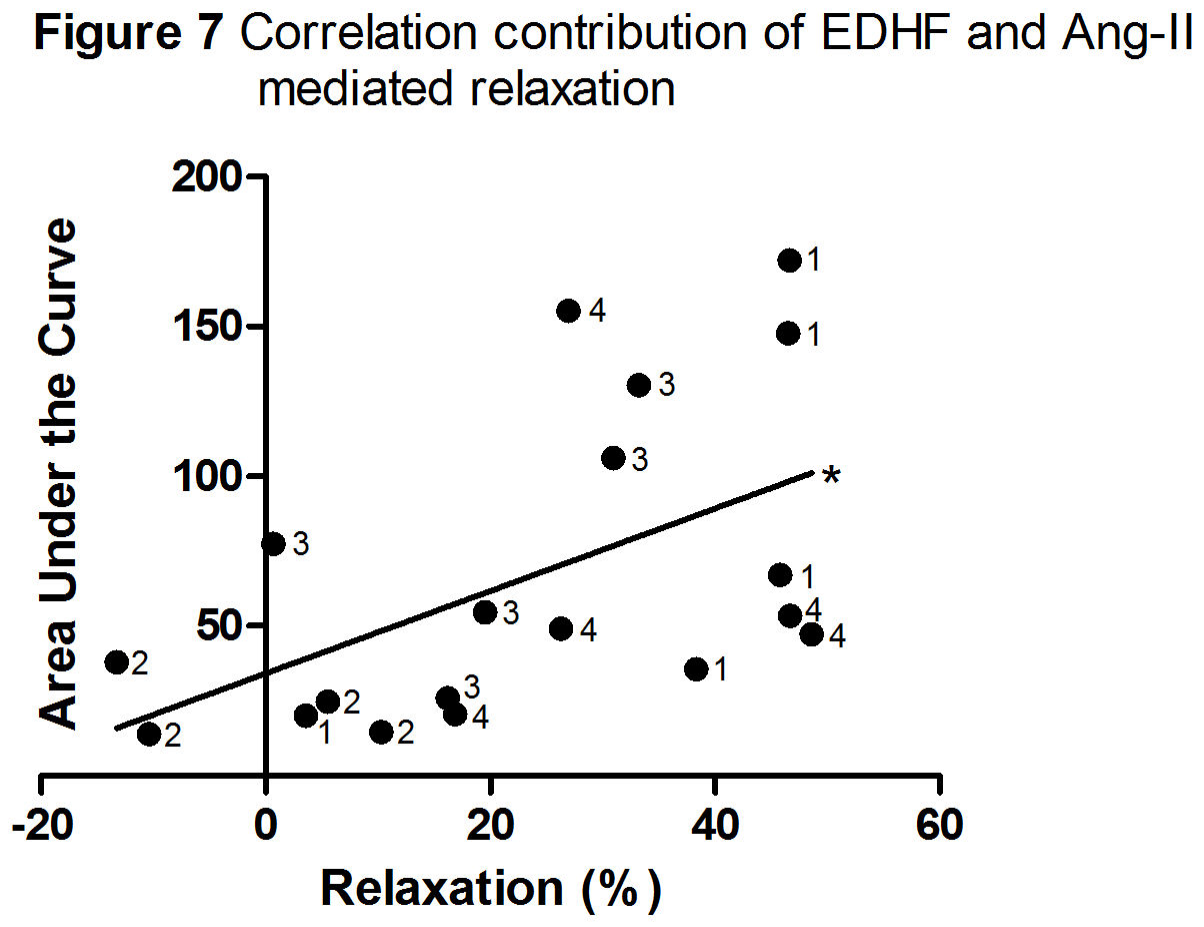
Correlation contribution of EDHF and Ang-II mediated relaxation through the AT2-R. The correlation between contribution of EDHF (AUC) and the Ang-II mediated relaxation through the AT2-R (relaxation (%)) in all groups. 1=non-pregnant-saline group; 2=pregnant-saline group; 3=pregnant-LPS group; 4=non-pregnant-LPS group. *:Pearson Correlation test, r=0.535, p<0.05.

The Ang-II relaxation mediated through the AT2-R significantly correlated with the contribution of EDHF (r=0.535, p=0.018) (Figure 7). No significant correlation was found between Ang-II mediated relaxation through the AT2-R and the contribution of NO (r=0.020, p=0.934; data not shown) and between Ang-II mediated relaxation through the AT2-R and the contribution of PG (r=0.411, p=0.081; data not shown). 

## Discussion

In this study, we investigated endothelial function by studying the endothelium-dependent relaxation and the role of the AT1-R and AT2-R in the Ang-II sensitivity of healthy control pregnant and preeclamptic rats [24] using aortic rings in an organ bath setup for isotonic displacement [40]. The model used for experimental preeclampsia, i.e. the low dose LPS-infused pregnant rat is an established model for preeclampsia, which has been used for many years [25,26]. It is not only characterized by hypertension and proteinuria in the last week of pregnancy [24,28], but also with endothelial cell activation and generalized inflammation [25]. Unfortunately, due to the experimental setup we were not able to measure blood pressure in this study. However, as stated above, the model used is an established model for studying preeclampsia and all studies performed, including recent studies in our lab, showed increase in blood pressure in this preeclampsia rat model.

We found that during pregnancy, there appeared to be a decreased role for contractile PG and a decreased role for EDHF in acetylcholine-induced relaxation of the aorta as compared to the non-pregnant state. We also found that the Ang-II sensitivity was blunted during pregnancy: we demonstrated a decreased response upon binding of Ang-II to the AT1-R (vasoconstriction) as well as upon binding to the AT2-R (vasorelaxing) in P-saline infused rats compared with NP-saline infused rats. Interestingly, similar to human preeclampsia, the pregnancy-induced changes in endothelium-dependent relaxation as well as in the decrease in contraction and relaxation response upon Ang-II were not observed in experimental preeclampsia in the present study. In fact, the endothelium-dependent relaxation and the responses upon Ang-II in experimental preeclamptic rats were comparable to those in the non-pregnant rats. 

 Incubation of aortic rings with vehicle showed no differences in relaxation between the four groups, suggesting that the total vascular vasoactive capacity of the aorta is not altered during normal pregnancy or during preeclampsia in the rat. This does not corroborate with some other studies [41–43], but it is in line with the study of Ballejo et al., who also showed that endothelium dependent relaxation in response to acetylcholine is not altered in late pregnancy [44]. It also agrees with various other studies showing that acetylcholine induced relaxation does not differ in non-pregnant vs late pregnant rats [45–47]. Differences in results may be due to strain differences [48]or to differences in timing of termination of pregnant or non-pregnant animals, differences in vascular bed used or differences in experimental methods. However, SNP treatment at the end of the experiments, showed a significantly attenuated relaxation in pregnant rats compared to non-pregnant rats (results not shown). This may indicate that during pregnancy, relaxation in the aorta is more dependent on endothelium-derived factors than in the non-pregnant state. Other models for preeclampsia, TNF infusion [49] or IL-6 infusion [50] in pregnant Sprague-Dawley rats did show decreased total vascular reactivity in the aorta as compared with control rats. These are, however, other models, in another rat strain, which may explain the differences. 

In our study, also the role of NO in the vasodilatory capacity of the aorta did not differ between pregnant and non-pregnant rats. Although this suggestion is not in line with some previous studies [51,52], it agrees with others [44,46]. This lack of differences in role of NO between the groups in our study is in accordance with the lack of differences in aortic iNOS or eNOS expression between the groups. Various studies have suggested a role for NO in the relaxation in pregnancy and the contraction in preeclampsia. Indeed, some studies showed that endogenous production of NO and eNOS mRNA are increased in pregnant rats [38,53]. Also *in-vivo* treatment of rats with L-NMMA induced a higher increase in blood pressure in pregnant as compared to non-pregnant rats suggesting an increased role for NO in vascular vasoactive responses in pregnant versus non-pregnant rats [54]. It may be suggested that our lack of difference in the role of NO in the aorta may be due to the fact that endothelial NO production during pregnancy may be enhanced spontaneously or in response to vasoconstricting agents, but not in response to vasorelaxing agents [44]. As suggested above, differences in responses may also be due to strain differences, since vascular responses to pregnancy are generally lower in Wistar rats as compared to Sprague-Dawley rats [48]. Also differences in vascular beds used may account for differences between studies, since the aorta, which is a conduit vessel, and largely depends on NO, may respond differently than a mesenteric vessel, which is a resistance vessel and depends to a much lesser extend on NO [55]. However, methodological differences or differences in timing of pregnancy may also play a role. 

In contrast to NO, the involvement of vasoactive PG in acetylcholine-induced relaxation responses appeared to change during pregnancy in the present study. Changes in PG in pregnancy have also been found by Bobadilla et al. [56], but not by others studies (including another study of Bobadilla et al. [52,57]. As described above, differences might be due to difference in strain used and methodological differences since Aloamaka et al. studied responses upon vasocontractile agents. In NP rats, inhibition of PG with indomethacin enhanced acetylcholine-induced relaxation, indicating the involvement of contractile PG in rats. However, this effect was absent in P-saline infused rats, suggesting that pregnancy was associated with a larger role of vasorelaxing PG, such as prostacyclin, in endothelium dependent relaxation. Alternatively, a decrease in contractile PG or receptor down regulation of the prostaglandin route during pregnancy may also be suggested. This observation is strengthened by the observation that precontraction with phenylephrine after incubation with indomethacin is enhanced in P-saline infused rats as compared with the other 3 groups of rats. These data are in line with the suggestion that vasodilatory PG may oppose the action of vasoconstrictors in pregnancy [58]. As incubation with indomethacin caused an increase in relaxation in P-LPS infused rats, this putative role of prostacyclin during pregnancy is blunted in experimental preeclampsia. With these results, our findings seem to be in line with results in human preeclampsia [59–63], which showed decreased prostacyclin production in preeclampsia versus normal pregnancy [64], as well as with other models of preeclampsia [65,66]. The altered involvement of vasoactive PG in acetylcholine-induced relaxation responses found in our study, appeared independent of regulation of COXs expression, since we found no differences in mRNA expression of COX-1 or COX-2. However, we take into account that mRNA expression is not a surrogate for protein expression or post-translational effects in target cells. 

The role of EDHF in endothelium-dependent relaxation was studied using concomitant incubation of the aortic rings with L-NMMA and indomethacin. This results in inhibition of NO and PG, therefore the resultant relaxation response is due to EDHF, or other unknown factors, such as hydrogen sulfide [67] by means of exclusion. EDHF is an endothelium-derived relaxing factor that causes vasorelaxation in association with vascular smooth muscle hyperpolarization [68]. The chemical identity of EDHF is uncertain [13]. In our study in aortic rings, EDHF or these other factors significantly contributed to acetylcholine-induced relaxation in all groups, but was of significantly of less importance in P-saline infused rats. Other studies comparing the role of EDHF during pregnancy found an increased role for EDHF in pregnancy [52,69]. However, these studies were performed in mesenteric arteries. Results may be different in humans, since EDHF was found to play a significant role in myometrial and subcutaneous arteries of pregnancy compared to preeclampsia [15,70]. This inconsistency in our rat model may also be explained by the fact that different arteries were used, since it is well known that EDHF has different vasoactive properties depending on the arteries studied [55,71]. Indeed, in rat mesenteric arteries the role of EDHF in relaxation appears to be increased during pregnancy [69]. 

To study the role of the AT1-R and AT2-R in the blunted responsiveness to Ang-II during pregnancy [16], we studied the *in-vitro* responsiveness of the AT1-R and AT2-R to Ang-II in the rat. The contractile response to Ang-II was dramatically decreased in P-saline infused rats as compared to the NP rats, which is in line with a decreased blood flow reducing effect of Ang-II during human pregnancy [16]. Our data also confirm previous studies in the rat [72,73]. Also, the increased contraction response to Ang-II in aortic rings of experimental preeclamptic rats as compared to P-saline infused rats appears to be in line with the well-known increased Ang-II sensitivity during human preeclampsia [19] and with studies in other models of experimental preeclampsia [74]. This increase in response to Ang-II may be caused by an increased AT1-R expression in LPS infused pregnant animals, although we only found a trend towards increase in AT1-R mRNA expression. Whether this increased response to Ang-II in the aorta of rats with experimental preeclampsia contributes to the hypertension seen in these animals, remains speculative, since the aorta is a conductance vessel and not a resistance vessel. Further studies into the response to Ang-II in other vessels on the preeclamptic rats are in progress. However, a role for Ang-II and the AT1-R in this model for experimental preeclampsia has been shown by Doering et al. who observed that hypertension was decreased in this model after treatment with the AT1-R antagonist losartan. 

Interestingly, we found that the vasorelaxing response to Ang-II mediated through the AT2-R was absent during late pregnancy in the P-saline infused rat and increased in the P-LPS infused rats, suggesting that also the AT2-R does play a role in the adaptations of the sensitivity to Ang-II during normal pregnancy and preeclampsia. In contrast to the present study, however, Stennett et al. observed an increased responsiveness of the AT2-R to Ang-II during normal pregnancy [38]. The difference between our study and the study by Stennett et al. may be explained by strain differences, since Stennett et al. used Sprague-Dawley rats rather than Wistar rats. A recent review of van Drongelen et al., showed large differences in pregnancy induced vascular responses between Wistar and Sprague-Dawley rats [48] or concomitant long-term use of AT1-R blockers as shown in another study [75]. Apart from placental and uterine tissue [76–78], data on function and expression of the AT1-R and AT2-R in tissues of humans during pregnancy are largely lacking. Therefore, the role of the AT2-R versus the AT1-R during human pregnancy and preeclampsia is relatively unclear. Since both the vasocontractory response (via the AT1-R) as well as relaxation response (via the AT2-R) to Ang-II were blunted at the end of pregnancy, but not in experimental preeclampsia, the RAAS may be of relative low importance in blood pressure control at the end of normal rat pregnancy, while in preeclamptic rats, the contribution of the RAAS may be enhanced. 

Finally, our results show that the decreased involvement of EDHF in acetylcholine-induced relaxation during pregnancy correlates with a decreased relaxation responsiveness of the AT2-R to Ang-II in these conditions. This correlation may be in line with the suggestion that bradykinin and the B2-receptor are involved in relaxation induced by Ang-II [79] and the notion that bradykinin-induced relaxation is typically mediated by multiple EDHF’s [80]. Although NO and PG-F2α are also suggested to play a role in the relaxation after binding of Ang-II to the AT2-R, we did not find a significant correlation between the role of NO or PG in the vasoactive capacity of the aorta and the AT2-R induced relaxation. This may suggest that the contribution of NO to relaxation was relatively constant and that differences in responses of the AT2-R to Ang-II during pregnancy and experimental preeclampsia may result from differences in the role of EDHF. The lack of correlation between the role of PG and the vasorelaxing effect of Ang-II may result from the fact that we did not specifically study PG-F2α, but PG in total. 

 Our observations were obtained with aorta which is a conductance vessel rather than a resistance vessel typically involved in blood pressure regulation. Our present observations in the aorta provide evidence of altered involvement of different endothelial mediators in acetylcholine-induced relaxation and responsiveness to Ang-II in pregnancy and preeclampsia. They need to be confirmed in future studies employing preparations of other vessels – such as small resistance arteries, for example – to more directly link changes in vascular function to hypertension in pregnancy. However, it may be speculated that such changes may play a role in the regulation of blood pressure by influencing vascular smooth muscle tone and therefore aortic stiffness and thus central blood pressure [81,82].It may be of additional interest in future studies to include the role of Ang 1-7 when aiming to unravel the RAAS pathways involved in the development of preeclampsia. Ang 1-7 is a metabolite of Ang-II which is able to counteract the vasoconstriction effect of Ang-II by binding to the MAS-receptor and subsequently causing vasodilation via NO through eNOS activation [83] .

From this study, the pathway of how LPS induced the endothelial dysfunction and increased Ang-II seen in this experimental rat model for preeclampsia is still unknown. It is interesting to note that although the infusion of LPS took place on day 14 of pregnancy, endothelial dysfunction was observed on day 20 of pregnancy. This implies that LPS, which is infused during 1hr on day 14, and cannot be traced in the circulation 15 minutes after infusion (unpublished data from previous work), induced a long-lasting effect in pregnant rats. This might be a direct effect of LPS on the endothelial cells, since LPS was shown to directly affect endothelial cells *in-vitro* [84]. However, it might also be an indirect effect, since it has been shown that LPS induced long-lasting activation of inflammatory cells in pregnant rats [26,30]. These activated inflammatory cells, for instance by producing oxygen free radicals, may then induce the endothelial cell dysfunction [85]. Future studies will be performed to test these two options. 

In conclusion, pregnancy in the rat was associated with a change in the involvement of different mediators of thoracic aortic endothelial relaxation function and the response to Ang-II. The contribution of vasodilatory PG to acetylcholine-induced relaxation was increased while that of EDHF was decreased in pregnant rats, as compared to that in non-pregnant rats. Moreover, we observed a decreased sensitivity to Ang-II (both contraction and relaxation) in the aorta during pregnancy in the rat. Interestingly, the pregnancy-induced changes appeared to be absent in experimental preeclampsia in the rat. The present findings may imply that the LPS-induced pregnant rat is a suitable model for future studies aiming to unravel the etiology of preeclampsia and test treatment options directed to Ang-II receptors and endothelial cells in preeclampsia. 
